# Rapamycin Does Not Compromise Exercise‐Induced Muscular Adaptations in Female Mice

**DOI:** 10.1111/acel.70183

**Published:** 2025-07-24

**Authors:** Christian J. Elliehausen, Szczepan S. Olszewski, Dennis M. Minton, Carolyn G. Shult, Aditya R. Ailiani, Michaela E. Trautman, Reji Babygirija, Dudley W. Lamming, Troy A. Hornberger, Adam R. Konopka

**Affiliations:** ^1^ Division of Geriatrics and Gerontology, Department of Medicine University of Wisconsin‐Madison Madison Wisconsin USA; ^2^ Geriatric Research Education and Clinical Center William S. Middleton Memorial Veterans Hospital Madison Wisconsin USA; ^3^ Division of Endocrinology, Department of Medicine University of Wisconsin‐Madison Madison Wisconsin USA; ^4^ William S. Middleton Memorial Veterans Hospital Madison Wisconsin USA; ^5^ University of Wisconsin‐Madison Comprehensive Diabetes Center Madison Wisconsin USA; ^6^ Wisconsin Nathan Shock Center of Excellence in the Basic Biology of Aging Madison Wisconsin USA; ^7^ Department of Comparative Biosciences University of Wisconsin‐Madison Madison Wisconsin USA; ^8^ School of Veterinary Medicine University of Wisconsin‐Madison Madison Wisconsin USA

**Keywords:** aging, exercise, glucose tolerance, hypertrophy, mTOR, muscle

## Abstract

An increasing number of physically active adults are taking the mTOR inhibitor rapamycin off label with the goal of extending healthspan. However, frequent rapamycin dosing disrupts metabolic health during sedentary conditions and abates the anabolic response to exercise. Intermittent once‐weekly rapamycin dosing minimizes many negative metabolic side effects of frequent rapamycin in sedentary mice. However, it remains unknown how different rapamycin dosing schedules impact metabolic, physical, and skeletal muscle adaptations to voluntary exercise training. Therefore, we tested the hypothesis that intermittent rapamycin (2 mg/kg; 1×/week) would avoid detrimental effects on adaptations to 8 weeks of progressive weighted wheel running (PoWeR) in adult female mice (5‐month‐old) by evading the sustained inhibitory effects on mTOR signaling by more frequent dosing schedules (2 mg/kg; 3×/week). PoWeR improved maximal exercise capacity, absolute grip strength, and myofiber hypertrophy with no differences between vehicle or rapamycin‐treated mice despite greater voluntary running volume with intermittent rapamycin treatment. Conversely, frequent and intermittent rapamycin‐treated mice had impaired glucose tolerance and insulin sensitivity compared to vehicle‐treated mice after PoWeR; however, intermittent rapamycin reduced the impact on glucose intolerance versus frequent rapamycin. Collectively, these data in adult female mice suggest that (1) rapamycin is largely compatible with the physical and skeletal muscle benefits of PoWeR and (2) the detrimental effects of rapamycin on glucose metabolism in the context of voluntary exercise may be reduced by intermittent dosing.

## Graphical abstract

Rapamycin is largely compatible with the physical and skeletal muscle benefits of progressive weighted wheel running (PoWeR). The detrimental effects of rapamycin on glucose metabolism in the context of voluntary exercise are mitigated by intermittent dosing.

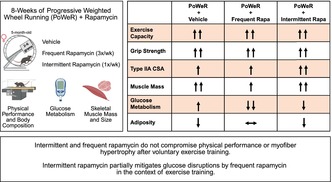

## 1 Introduction

Rapamycin (sirolimus), a mechanistic target of rapamycin (mTOR) protein kinase inhibitor, is an FDA‐approved drug that can extend lifespan in multiple model systems (Mannick and Lamming 2023). In inbred strains of mice, rapamycin can extend lifespan even when started early in life (4 months of age or earlier) (Anisimov et al. 2011; Fok et al. 2014; Neff et al. 2013). In genetically heterogeneous UM‐HET3 mice, lifespan extension from rapamycin is influenced by dose, sex, and age at treatment initiation, where rapamycin may have greater lifespan extension when administered at higher doses, earlier in life, and in females (Strong et al. 2020; Miller et al. 2014; Harrison et al. 2009). These results have led to the hypothesis that starting rapamycin earlier in adulthood may confer the greatest lifespan‐extending effects (Blagosklonny 2022). In addition to lifespan, rapamycin delays several age‐related pathologies, including preservation of physical and skeletal muscle function in sedentary mice and rats to combat sarcopenia and frailty (Ham et al. 2020; Joseph et al. 2019).

Despite the positive effects on lifespan and many indices of healthspan, prolonged treatment with rapamycin is associated with dose‐dependent risks of metabolic side effects, including glucose intolerance, insulin resistance, and dyslipidemia (Johnston et al. 2008; Bissler et al. 2017). Rapamycin acutely and potently inhibits mTOR complex 1 (mTORC1) while prolonged rapamycin treatment can inhibit mTOR complex 2 (mTORC2) signaling in culture and mice (Ye et al. 2012; Lamming et al. 2012; Sarbassov et al. 2006). A leading model suggests that inhibition of mTORC1 mediates the geroprotective effects of rapamycin, while inhibition of mTORC2 signaling has pernicious effects on metabolic health, frailty, and survival in mice (Mannick and Lamming 2023; Lamming et al. 2014; Mizunuma et al. 2014; Apelo et al. 2020; Chellappa et al. 2019; Yu et al. 2019). Intermittent rapamycin dosing strategies more selectively inhibit mTORC1 and extend lifespan in female mice, while circumventing metabolic side effects through reduced mTORC2 inhibition (Arriola Apelo, Neuman, et al. 2016; Arriola Apelo, Pumper, et al. 2016). Due to these exciting data, an increasing number of physically active adults are now prophylactically taking rapamycin, largely using intermittent dosing schedules, even though the impact of rapamycin on the health benefits of regular physical activity and human healthspan remains unknown (Kaeberlein et al. 2023).

Preclinical and prospective studies in humans suggest engaging in either endurance and/or resistance activity is one of the most potent stimuli to protect against multimorbidity and premature mortality (Holloszy 1997; Zhao et al. 2020). Furthermore, improvements in cardiorespiratory fitness, skeletal muscle function, and insulin sensitivity that are traditional adaptations of endurance and/or resistance exercise training are associated with decreased risk of morbidity and mortality (Abou Sawan et al. 2023; Imboden et al. 2018; Lanza et al. 2008).

To model adaptations commonly observed after combined endurance and resistance exercise in humans, we and others have recently implemented progressive weighted wheel running (PoWeR) in adult and aged mice (Dungan et al. 2019, 2022; Englund et al. 2020). PoWeR is a voluntary, high‐volume exercise paradigm that increases muscle fiber size and oxidative capacity in multiple muscles while improving whole‐body glucose metabolism and physical function. We have previously demonstrated that short‐term PoWeR in adult mice stimulates mTORC1 signaling as evident by a 2–3‐fold increase in the phosphorylation of downstream substrates p70S6K (T389) and rpS6 (S235/236) particularly in muscles that increase mass after longer‐term PoWeR (Elliehausen et al. 2025).

mTORC1 signaling is a critical regulator of skeletal muscle anabolism whereby disrupting mTORC1 signaling genetically or by rapamycin attenuates the muscle protein synthetic and hypertrophic response to exercise in rodents and humans (Ogasawara et al. 2016; Bodine et al. 2001; Drummond et al. 2009; Gundermann et al. 2014; Goodman et al. 2011; Philp et al. 2015; You et al. 2019). Similarly, genetic knockout of *Rictor*, a core component of mTORC2, reveals blunted exercise‐induced glucose uptake and muscle protein synthesis (Kleinert et al. 2017; Ogasawara et al. 2020). Improved functional adaptations to muscle mass, hypertrophy, insulin sensitivity, and physical performance are associated with repeated, increased signaling through mTORC1/2 (Egan and Sharples 2023).

Collectively, these data support the current status quo that rapamycin‐mediated inhibition of mTORC1/2 is largely contraindicated with exercise. However, the majority of preclinical studies to date have only provided rapamycin frequently at high doses or on the same day as the exercise stimuli and have primarily evaluated the effects on the protein synthetic and hypertrophic responses to models of simulated resistance or acute forced treadmill exercise (Ogasawara et al. 2016; Goodman et al. 2011; Philp et al. 2015). It is also unknown whether inhibition of mTORC1 by rapamycin during a physiological, voluntary, and high‐volume model of exercise impairs systemic and skeletal muscle adaptations and whether these effects vary with different rapamycin dosing schedules.

Therefore, the goals of this study were to (1) identify if mTOR inhibition via rapamycin will alter the physical, metabolic, and skeletal muscle adaptations to PoWeR in adult female mice and (2) determine whether there are dosing regimen‐dependent outcomes that would support or refute the concomitant use of rapamycin and exercise in future healthspan and lifespan studies. Adult female mice were a priori selected for the current study because the lifespan‐extending effects of rapamycin may be relatively greater (1) when started earlier versus later in life and (2) in females compared to males. Furthermore, female mice run more consistently and greater volumes than males (Mannick and Lamming 2023; Miller et al. 2014; Konhilas et al. 2004). Here, we demonstrate that frequent (3×/week) and intermittent (1×/week) rapamycin dosing are compatible with the physical performance and muscle hypertrophy benefits of PoWeR. However, we identify frequent rapamycin disrupts whole‐body glucose homeostasis during PoWeR, which is reduced by intermittent dosing. These results provide support for concomitant rapamycin and exercise interventions aimed at evaluating lifespan and healthspan in aged mice, although alternative dosing regimens may be warranted to further minimize metabolic disruptions.

## 2 Results

### 2.1 Time Course of Rapamycin on Skeletal Muscle mTORC1 Signaling

Previous reports in mice indicate that rapamycin (1.5–2.0 mg/kg) inhibits skeletal muscle rpS6 phosphorylation, a downstream effector of mTORC1 signaling, for up to 48 h and returns toward baseline levels by ~60 h (D'Hulst et al. 2022; Arriola Apelo, Neuman, et al. 2016). To confirm these findings and gain insight into whether frequent rapamycin (3×/week) persistently inhibits mTORC1 while intermittent rapamycin (1×/week) transiently inhibits mTORC1 signaling between weekly doses, we performed a time course experiment in a separate cohort of age‐matched female mice receiving 4 weeks of once weekly vehicle or rapamycin (i.p. 2 mg/kg). Compared with vehicle control, rapamycin inhibited skeletal muscle rpS6 phosphorylation at 24 and 48 h after the last injection but returned to baseline by 72 h (Figure S1A). Therefore, these data support our objective of determining how persistent or transient inhibition of mTORC1 signaling by different rapamycin dosing schedules would impact whole‐body and skeletal muscle adaptations to voluntary exercise training.

### 2.2 Influence of Rapamycin on Running Behavior and Body Composition After PoWeR

**FIGURE 1:**
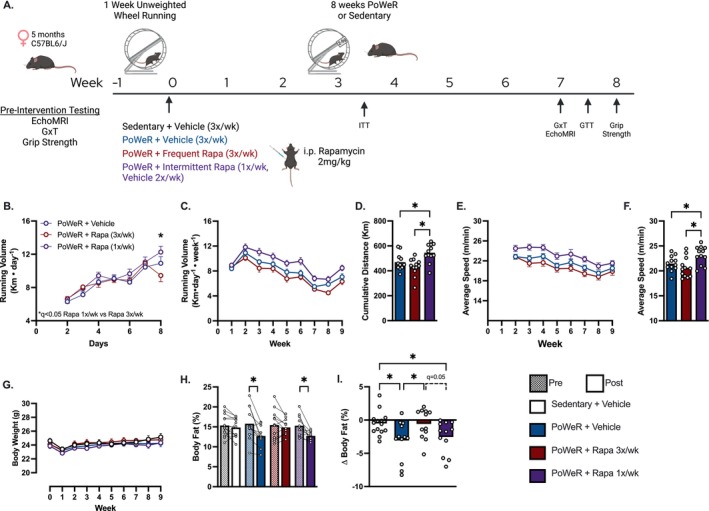
Physiological characterization. (A) Study schematic and timepoints for key measurements. (B) Daily distance run during unweighted acclimation (Days 2–7) leading up to the first day of rapamycin or vehicle injections (Day 8). (C) Average daily voluntary running distance per week and (D) cumulative running volume. (E, F) Average running speed during PoWeR. (G) Weekly bodyweight. (H) Body fat percentage pre and post intervention. (I) Delta (post minus pre) body fat percentage. *N* = 11–14 per group. Data presented as mean plus individual data points or error bars represent SEM. Data analyzed by two‐way ANOVA with repeated measures to assess main effects for time, treatment, and time × treatment interactions, and multiple post hoc comparisons were FDR corrected with a two‐stage step‐up method of Benjamini, Krieger, and Yekutieli (B, E–G) otherwise one‐way ANOVA with multiple comparisons FDR corrected by a two‐stage step‐up method (C, H). *q < 0.05. Panel A created in BioRender.com. GTT, glucose tolerance test; GxT, graded exercise test; ITT, insulin tolerance test.

Following preintervention testing, 5‐month‐old female mice were acclimated to an unweighted running wheel for 1 week prior to allocation to either continue PoWeR or remain sedentary for the remaining 8 weeks of the intervention (Figure 1A). Mice allocated to PoWeR were randomized to minimize baseline running volume differences and began either rapamycin (3×/week or 1×/week) or vehicle (3×/week) injections. During the first week of unweighted wheel running, there were no differences in running volume between groups. We next sought to determine if different dosing schedules of rapamycin altered spontaneous running behavior during PoWeR. We identified a divergence in running behavior between intermittent rapamycin versus frequent rapamycin starting after the first dose of intermittent rapamycin that continued throughout the intervention (Figure 1B–D). Since the frequent rapamycin‐treated mice did not increase running volume after their first dose, these findings suggest the greater running volume with intermittent rapamycin may not be directly related to rapamycin. The greater running volume with intermittent rapamycin (Rapa 1×/week) appeared to be due to mice self‐selecting a higher running speed compared to mice treated with vehicle or frequent rapamycin (Figure 1E,F). To understand the impact of running volume on outcomes presented next, we performed linear regression analyses (Figure S2). Running volume was positively associated with increased adiposity in all mice, which is counterintuitive and does not support that the greater running volume in mice treated with intermittent rapamycin contributed to greater loss of adiposity. Running volume was not significantly associated with any other outcome, suggesting the differences in outcomes presented herein are primarily influenced by rapamycin dosing schedules.

Weekly assessment of bodyweight did not differ between groups over the course of the intervention (Figure 1G) however, PoWeR trained mice treated with vehicle or intermittent rapamycin reduced adiposity, while mice treated with frequent rapamycin did not (Figure 1H,I).

### 2.3 Disruptions to Glucose Metabolism With Frequent Rapamycin During PoWeR Are Reduced by Intermittent Rapamycin

Impaired glucose homeostasis is a negative metabolic side effect of frequent administration of rapamycin or rapalogs in sedentary humans (Johnston et al. 2008; Bissler et al. 2017). In mice, disruptions to glucose metabolism can be observed as early as 2 weeks of rapamycin treatment (Lamming et al. 2012; Yang et al. 2012). Therefore, to determine the impact of rapamycin on glucose metabolism after PoWeR, we evaluated fasting glucose, glucose tolerance, and insulin tolerance after 3 or 7 weeks following the onset of rapamycin treatment (Figure 1A). All tolerance tests were performed 24 h after the last exercise bout and 24 or 72 h after the last frequent and intermittent rapamycin dose, respectively. 72 h was selected for the intermittent group as this represents the timing for when mTORC1 inhibition is alleviated in skeletal muscle in sedentary mice receiving intermittent rapamycin (2 mg/kg, 1×/week) (Figure S1) (Arriola Apelo, Neuman, et al. 2016).

**FIGURE 2:**
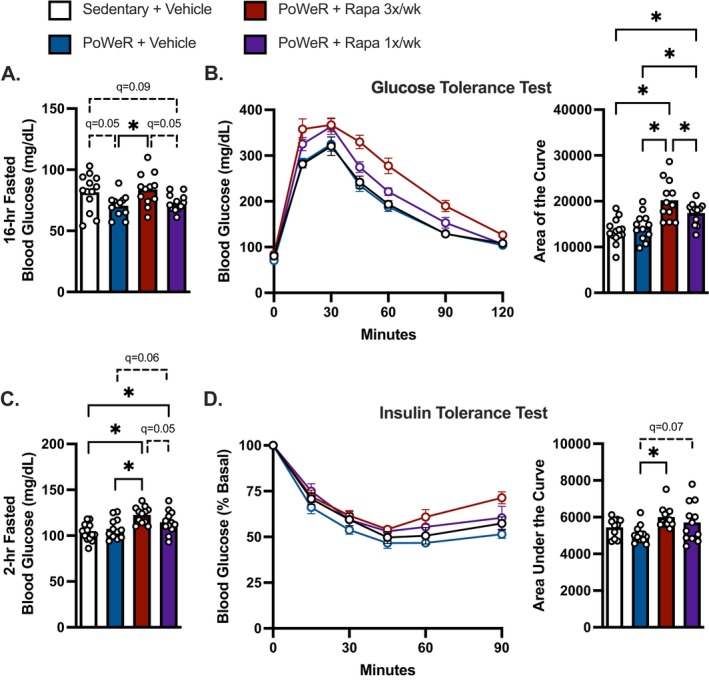
Disruptions to glucose homeostasis by frequent rapamycin are less severe by once weekly intermittent dosing during PoWeR. (A) Overnight fasted (~16‐h) blood glucose level prior to glucose injection. (B) Blood glucose curve during a glucose tolerance test and area of the curve (AOC) calculated as the area under the curve relative to starting blood glucose value prior to glucose injection. (C) Fasted (2‐h) blood glucose prior to insulin injection. (D) Blood glucose curve during an insulin tolerance test and area under the curve (AUC). *N* = 11–14 per group. Data presented as mean plus individual data points or error bars represent SEM. Data analyzed by one‐way ANOVA with multiple comparisons FDR corrected by a two‐stage step‐up method (A–D). *q < 0.05.

Following an overnight fast (~16‐h) PoWeR‐trained mice treated with vehicle had lower (*q* = 0.05) fasting blood glucose compared with sedentary control (Figure 2A). PoWeR‐trained mice treated with frequent rapamycin had greater fasting blood glucose compared with vehicle and intermittent rapamycin and were not different from sedentary mice (Figure 2A). Frequent and intermittent rapamycin both disrupted glucose tolerance after PoWeR compared with vehicle control, as evidenced by a 40% and 21% greater glucose burden determined by the area of the blood glucose curve (Figure 2B). However, glucose intolerance was less severe in intermittent versus frequent rapamycin‐treated mice after PoWeR (Figure 2B).

Following a 2‐h fast and prior to the insulin tolerance test, frequent and intermittent rapamycin treatments resulted in elevated fasting glucose levels compared with sedentary controls (Figure 2C). After PoWeR, mice treated with frequent but not intermittent rapamycin (*q* = 0.07) were relatively less insulin sensitive compared to vehicle‐treated mice determined by the area under the relative (% Basal) blood glucose curve (Figure 2D). Collectively, these data suggest the disruptions to glucose metabolism with frequent rapamycin treatment may be less severe with intermittent rapamycin compared to frequent rapamycin during PoWeR.

### 2.4 Rapamycin Does Not Attenuate the Improvement in Whole‐Body Physical Capacity nor Myofiber Hypertrophy After PoWeR

**FIGURE 3:**
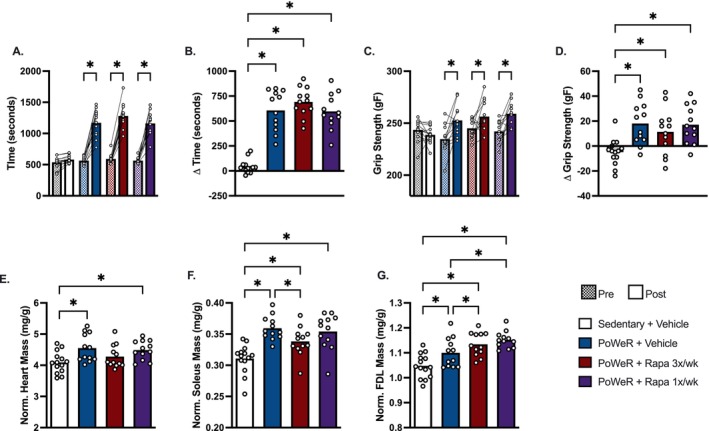
Rapamycin does not impair physical performance adaptations to PoWeR. (A) Time run during a graded exercise test. (B) Delta (post minus pre) time run. (C) All‐limb grip strength. (D) Delta (post minus pre) all‐limb grip strength (E) Heart mass normalized to body weight. (F) Soleus muscle mass normalized to body weight. (G) FDL muscle mass normalized to body weight. *N* = 12–14/group. Data presented mean plus individual data points. Data analyzed by two‐way ANOVA with repeated measures was used to assess main effects for time, treatment, and time × treatment interactions, and multiple post hoc comparisons were FDR corrected with a two‐stage step‐up method of Benjamini, Krieger, and Yekutieli (A, C) otherwise one‐way ANOVA with multiple comparisons FDR corrected by a two‐stage step‐up method (B, D–G). *q < 0.05.

We next aimed to determine whether rapamycin would alter the physical performance benefits and skeletal muscle hypertrophy from PoWeR. Despite differences in running volume and body composition after PoWeR between vehicle, intermittent, and/or frequent rapamycin treated mice, rapamycin did not influence the increase in maximal running capacity nor absolute all‐limb grip strength after PoWeR (Figure 3A–D).

Considering the notion that inhibition of mTORC1 by rapamycin would blunt muscle hypertrophy, we evaluated mass and/or fiber type‐specific cross‐sectional area (CSA) in the heart, soleus, and flexor digitorum longus (FDL) which we and others have identified to respond to PoWeR (Dungan et al. 2019, 2022; Englund et al. 2020; Elliehausen et al. 2025). Frequent but not intermittent rapamycin attenuated the increase in heart mass normalized to body mass after PoWeR (Figure 3E), although the impact on heart mass by frequent rapamycin did not seem to significantly impact maximal exercise performance. PoWeR increased soleus and FDL muscle mass normalized to body mass in vehicle, frequent, and intermittent rapamycin treated mice (Figure 3F,G). However, we identified muscle specific effects of rapamycin on muscle mass gains after PoWeR. In the oxidative soleus, frequent but not intermittent rapamycin treated mice had lower muscle mass after PoWeR compared with vehicle control (Figure 3F). Conversely, in the more glycolytic FDL, intermittent and frequent rapamycin had greater muscle mass after PoWeR compared with vehicle control (Figure 3G).

**FIGURE 4:**
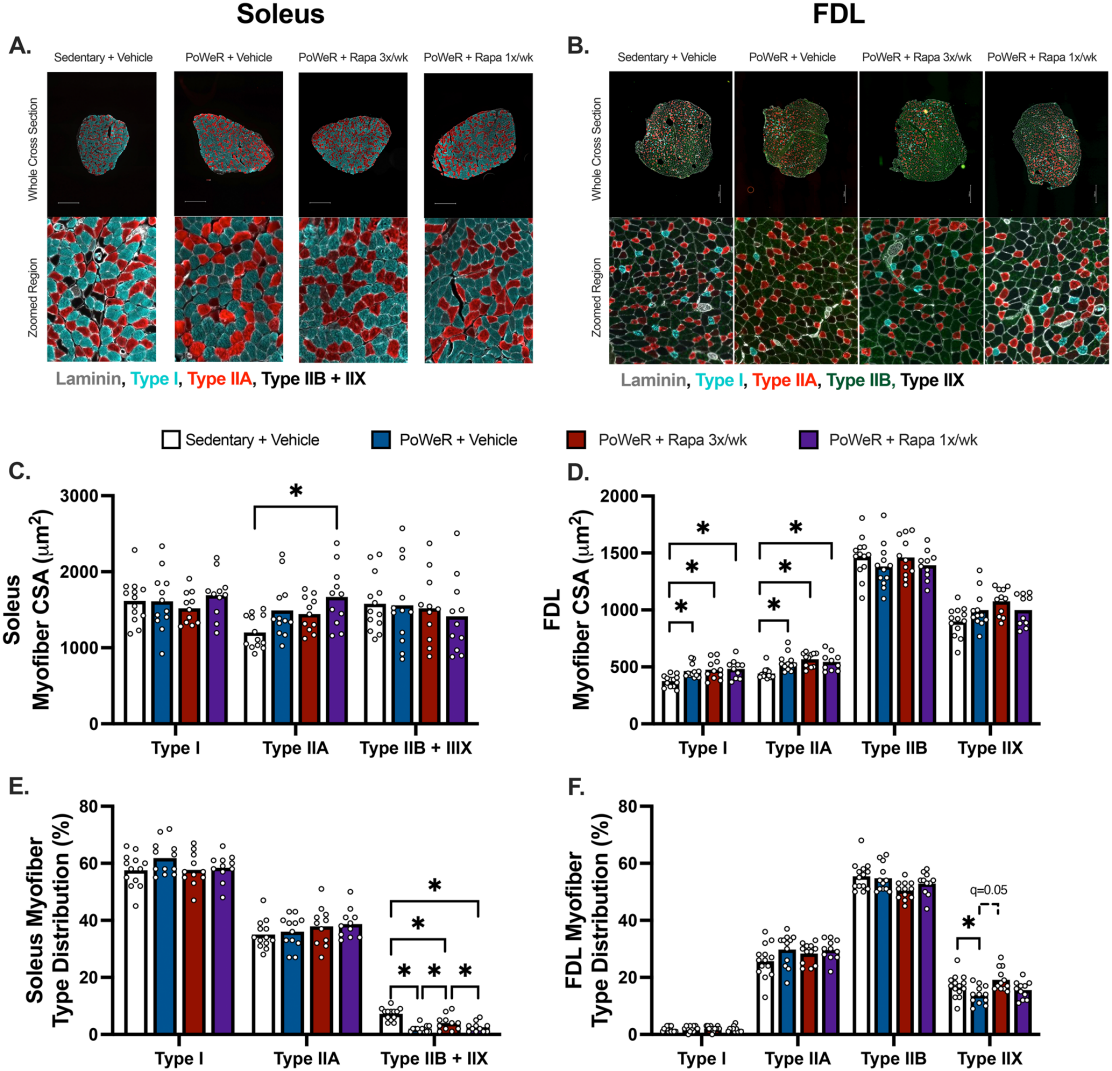
Myofiber hypertrophy in the soleus and FDL after PoWeR is not impeded by rapamycin. Representative mid‐belly cross sections of the (A) soleus and (B) FDL subjected to immunohistochemistry for myofiber typing (Type I, Type IIA, Type IIB, and Type IIX). Due to low abundances of IIB and IIX myofibers in the soleus, these fiber types were pooled. Average myofiber size per myofiber type in the (C) soleus and (D) FDL. Myofiber type distribution in the (E) soleus and (F) FDL. *N* = 11–13 per group. Data presented as mean plus individual data points. Each myofiber type was analyzed by one‐way ANOVA with multiple comparisons FDR corrected by a two‐stage step‐up method (C–F). *q < 0.05. Scale bar in A, B = 500 μm.

Performing immunohistochemistry on whole muscle cross sections permitted the evaluation of fiber type‐specific CSA in the soleus and FDL (Figure 4A,B). In the soleus, PoWeR‐trained mice treated with intermittent rapamycin had ~40% greater Type IIA CSA compared with sedentary controls, while PoWeR‐trained mice treated with vehicle (+24%, *q* = 0.07) or frequent rapamycin (+20%, *q* = 0.1) had nonsignificantly greater Type IIA CSA compared with sedentary (Figure 4C). In the FDL, PoWeR‐trained mice had greater Type I and IIA myofiber CSA compared to sedentary, with no differences detected between PoWeR‐trained groups (Figure 4D).

All PoWeR‐trained mice, independent of rapamycin treatment, had a lower proportion of Type IIB + IIX myofibers in the soleus compared with sedentary (Figure 4E). However, in PoWeR‐trained mice, the proportion of IIB + IIX myofibers was greater with frequent rapamycin than intermittent rapamycin and vehicle. Myofiber type distribution in the FDL was largely unaffected by PoWeR with or without rapamycin treatment, with only the frequent rapamycin group having a small but statistically significant greater proportion of FDL Type IIX fibers compared to vehicle (Figure 4F). These results confirm PoWeR increases Type I and/or IIA fiber size while rapamycin did not negatively impact myofiber hypertrophy after PoWeR. These data also highlight a discrepancy between muscle mass and myofiber size, urging caution when interpreting mass increases as muscle hypertrophy.

### 2.5 Skeletal Muscle mTOR Signaling After PoWeR

**FIGURE 5:**
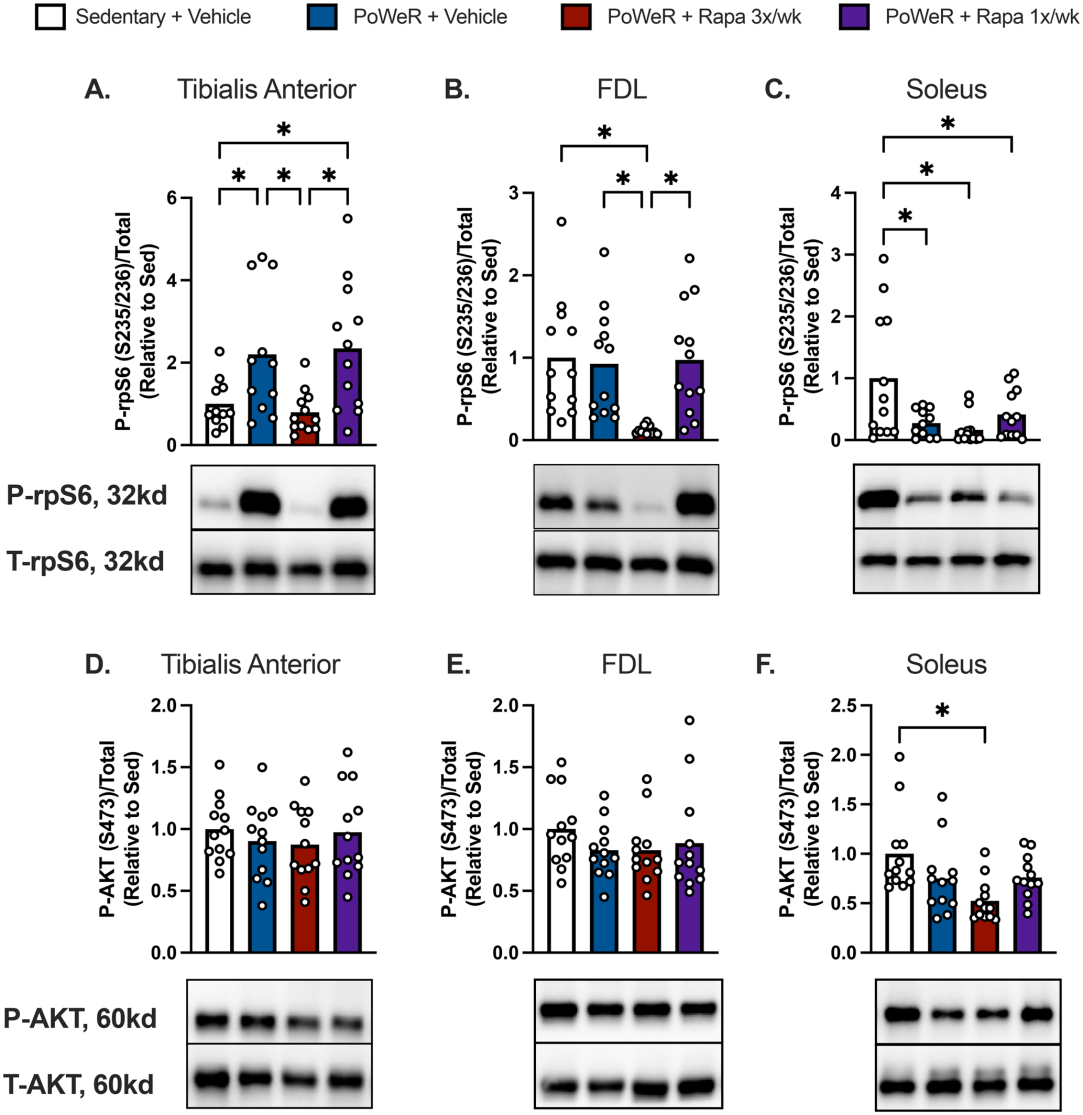
Skeletal muscle mTORC1/2 signaling following PoWeR training and/or rapamycin. Ratio of phosphorylated rpS6 (S235/236) to total protein via immunoblotting in the (A) tibialis anterior, (B) FDL, and (C) soleus with reflective immunoblot panels below corresponding to the treatment group order in the figures. Ratio of phosphorylated AKT (S473) to total protein via immunoblotting in the (D) tibialis anterior, (E) FDL, and (F) soleus. *N* = 11–12 per group. Data presented as mean plus individual data points. Data analyzed by one‐way ANOVA with multiple comparisons FDR corrected by a two‐stage step‐up method (A–F). *q < 0.05.

Finally, we aimed to evaluate mTORC1 and mTORC2 signaling in multiple skeletal muscles to associate with the observed physiological and skeletal muscle outcomes. Skeletal muscles were collected following a 24‐h running wheel lock and 2‐h fast. The last dose of frequent rapamycin was provided 24 h prior to tissue collection, while the intermittent rapamycin was dosed 7 days prior. We evaluated phosphorylation of ribosomal protein S6 (rpS6 S235/236), a downstream target of mTORC1 signaling, in the tibialis anterior (TA), soleus, and FDL. In the TA, greater phosphorylation of rpS6 was observed in PoWeR‐trained mice treated with vehicle or intermittent rapamycin. Conversely, phosphorylation of rpS6 was no different between PoWeR‐trained mice treated with frequent rapamycin versus sedentary controls (Figure 5A). In the FDL, frequent rapamycin suppressed the phosphorylation of rpS6 compared to both PoWeR‐trained vehicle and intermittent rapamycin groups, as well as sedentary controls (Figure 5B). In the soleus, we found all PoWeR‐trained groups had lower phosphorylation of rpS6 compared with sedentary controls (Figure 5C). These results are consistent with the expected kinetics of rapamycin induced suppression of rpS6 in skeletal muscle.

Since disruption to mTORC2 signaling is a proposed mechanism for metabolic dysfunction with prolonged rapamycin treatment, we probed for phosphorylated AKT (S473) as a substrate of mTORC2. We identified no differences in the phosphorylation of AKT between any groups in the TA or FDL (Figure 5D,E). However, in the soleus, we did detect lower AKT in PoWeR‐trained mice treated with frequent rapamycin compared with sedentary controls (Figure 5F).

## 3 Discussion

While exercise and rapamycin can independently improve multiple biological and physiological indices of aging, it was assumed that rapamycin and exercise are incompatible due to their opposing effects on mTOR signaling (Elliehausen et al. 2023). To address this fundamental question, the primary goals of this study were to (1) determine the influence of rapamycin on mTOR signaling and cellular and physiological adaptations to PoWeR and (2) whether these responses differed depending on dosing schedule in adult female mice. The major findings from this study were that despite differences in dosing frequency, neither frequent nor intermittent rapamycin undermined the improvement in physical capacity and myofiber hypertrophy by PoWeR. Frequent and intermittent rapamycin impaired glucose tolerance during PoWeR; however, the detrimental effects of rapamycin on glucose homeostasis were reduced by once weekly intermittent dosing. These data suggest that rapamycin is largely compatible with the majority of the health benefits of PoWeR and that intermittent once weekly dosing may be an approach to partially mitigate negative metabolic side effects of rapamycin during PoWeR in adult female mice.

### 3.1 Metabolic Function

Frequent administration of rapamycin at doses consistent with the FDA label increases risk for glucose intolerance and insulin resistance, which are known risk factors for cardiometabolic disease and would appear counter to healthy aging (Johnston et al. 2008; Bissler et al. 2017). Furthermore, inhibition of mTORC1 and/or mTORC2 signaling attenuates the acute increase in muscle protein synthesis and glucose uptake after a single bout of exercise and diminishes muscle hypertrophy in rodent models of resistance exercise (Ogasawara et al. 2016, 2020; Goodman et al. 2011; Ogasawara and Suginohara 2018). Despite these undesirable side effects, a growing number of people, particularly those younger to middle‐aged, are prophylactically consuming rapamycin off label with the intent to delay age‐associated conditions, in lieu of several ongoing human clinical trials testing if different rapamycin dosing strategies can safely and effectively improve the biology and physiology of aging and age‐associated diseases (Konopka and Lamming 2023). However, it remained unknown if rapamycin would alter the multitude of health benefits, like glycemic control, from voluntary exercise.

Here we show that despite engaging in a high‐volume exercise paradigm, mice treated with frequent rapamycin were glucose intolerant and insulin resistant compared to vehicle control. Importantly, intermittent once‐weekly dosing reduced the severity of impairments to whole‐body glucose homeostasis by frequent rapamycin after PoWeR but did not completely protect against glucose intolerance relative to vehicle control. Previous reports identified intermittent dosing once every 5 or 7 days prevents glucose intolerance and reduces pyruvate intolerance observed with more frequent rapamycin dosing (Arriola Apelo, Neuman, et al. 2016; Arriola Apelo, Pumper, et al. 2016). While our findings are largely in agreement with previous findings in sedentary mice whereby intermittent rapamycin reduced metabolic disruptions observed with frequent rapamycin, intermittent rapamycin still imparted some glucose intolerance relative to PoWeR‐trained mice treated with vehicle. An important distinction between the current study with PoWeR and the previous work in sedentary mice is the time of glucose tolerance testing relative to the last dose of rapamycin. Here we performed glucose tolerance testing 3 days after the last dose while the prior studies completed glucose tolerance testing 5 or 7 days after the last dose (Arriola Apelo, Neuman, et al. 2016). Regardless, the findings from this study are in line with the notion that intermittent dosing strategies may be effective at mitigating glucose disruptions by more frequent rapamycin even in the context of PoWeR.

### 3.2 Physiological Adaptations

Our data indicate that rapamycin does not impede engagement in voluntary exercise or impact the increase in exercise capacity after PoWeR. These results are largely supported by previous findings where a single dose of rapamycin did not impact the increase in mitochondrial protein synthesis after acute treadmill exercise, and frequent rapamycin treatment did not compromise exercise capacity nor spontaneous physical activity in sedentary mice (Ham et al. 2020; Philp et al. 2015; Ye et al. 2013). Cardiorespiratory fitness or VO_2_ max is one of the strongest predictors of morbidity and mortality in humans (Imboden et al. 2018; Mandsager et al. 2018). While we measured treadmill exercise capacity as a surrogate for cardiorespiratory fitness, the lack of impact by rapamycin on the improvement in exercise capacity after PoWeR is promising for the goal of reducing the risk of all‐cause mortality and improving healthy longevity.

Interestingly, frequent rapamycin appeared to prevent the decline in adiposity observed with PoWeR in the vehicle and intermittent rapamycin mice. These results are paradoxical to previous reports suggesting rapamycin decreased adiposity in sedentary animals, particularly in diet‐induced obesity models (Houde et al. 2010). Alternatively, models of mTORC1 activation also reduce adiposity (Magdalon et al. 2016). There may be a tightly regulated range of mTORC1/2 signaling necessary to regulate adiposity in the context of exercise training that frequent rapamycin treatment may disrupt, though this remains to be elucidated.

### 3.3 Muscle Mass, Size, and Grip Strength

We hypothesized frequent rapamycin treatment would mitigate the increase in skeletal muscle mTORC1 signaling, mass, hypertrophy, and grip strength after PoWeR. This hypothesis was supported by previous findings by our laboratory and others which have shown that PoWeR induced mTORC1 signaling, muscle hypertrophy, and improved whole‐body physical performance (Dungan et al. 2019, 2022; Englund et al. 2020; Elliehausen et al. 2025) while inhibition of mTORC1 signaling by high doses of rapamycin attenuated the muscle protein synthesis response to a single bout of resistance exercise in humans (Drummond et al. 2009; Gundermann et al. 2014) and muscle hypertrophy after nonvoluntary models of resistance exercise in rodents (Ogasawara et al. 2016; Goodman et al. 2011).

Consistent with other reports, our dosing schedule was sufficient to suppress rpS6 phosphorylation in skeletal muscle up to 48 h after the last dose. We interpret these data to indicate that frequent rapamycin dosing (3×/week) would largely inhibit mTORC1 over the course of the intervention, while intermittent (1×/week) dosing would remain transitory between doses. In line with this interpretation, frequent but not intermittent rapamycin suppressed downstream mTORC1 signaling after PoWeR in the TA and FDL collected 24 h after the last frequent rapamycin dose and 7 days after the last intermittent rapamycin dose.

While frequent rapamycin appeared to attenuate the increase in soleus mass after PoWeR, it did not compromise myofiber hypertrophy in the soleus or FDL, nor the improvement in whole‐limb grip strength after PoWeR. These data may suggest rapamycin‐sensitive mTORC1 signaling may be dispensable for the performance and hypertrophic adaptations to PoWeR. It is possible alternative signaling such as mTORC2, the mitogen‐activated protein kinase (MAPK) pathway, or the transcription factor MYC compensate for mTORC1 inhibition and/or serve as the primary drivers of PoWeR‐induced muscle hypertrophy (Ogasawara et al. 2019; Steinert et al. 2021; Murach et al. 2022). Alternatively, the residual amount of mTORC1 signaling observed in rapamycin‐treated mice after PoWeR may be sufficient to support myofiber hypertrophy. While the precise molecular transducers of exercise‐induced adaptations to PoWeR remain to be elucidated, our data introduce the notion that both transient and persistent mTORC1 inhibition via rapamycin do not impair skeletal muscle adaptations to 8 weeks of voluntary exercise in adult female mice.

### 3.4 Limitations and Considerations

In the current study, we chose to first study female mice because they have more consistent running behavior during voluntary wheel running and relatively greater lifespan extension by rapamycin compared with males (Mannick and Lamming 2023; Miller et al. 2014; Konhilas et al. 2004). We also prioritized female mice, which are traditionally underrepresented in exercise research. However, additional studies will be necessary to directly compare sex differences in response to exercise and rapamycin interventions.

While oral administration is most commonly reported by humans, we selected for i.p. dosing to standardize rapamycin administration while minimizing stress imparted by frequent oral gavage. Despite differences in pharmacokinetics between oral and i.p. dosing regimens, both administration routes have extended lifespan and improved conditions associated with aging (Mannick and Lamming 2023).

Further, we randomized mice matched for running volume as shown by equivalent running volumes in each group during the first week. Despite these efforts, PoWeR‐trained mice receiving intermittent rapamycin accumulated greater running volume than mice treated with vehicle or frequent rapamycin. While linear regression models indicate that running volume was not significantly associated with the outcomes presented here, future studies should strive to match running volume to avoid potential confounding variables.

Lastly, while the primary aim of the current study was to determine whether the PoWeR‐induced adaptations are influenced by different rapamycin dosing regimens, the omission of sedentary groups treated with rapamycin precludes our ability to assess the interaction between rapamycin and exercise. Rapamycin or its analogs at high and frequent doses impair glucose tolerance in sedentary rodents, but whether this is mitigated or exacerbated by concurrent exercise training remains to be elucidated.

## 4 Conclusion

mTORC1 is considered a central regulator of exercise adaptations. Since inhibition or ablation of mTORC1 was previously shown to attenuate the increase in muscle protein synthesis and muscle hypertrophy after muscle contractions, the dogma was that rapamycin may obstruct the health benefits of exercise training despite never being formally tested. In contrast, the data presented here indicate that combining rapamycin with 8 weeks of voluntary exercise in adult female mice is generally compatible with physical performance and skeletal muscle adaptations. However, more frequent dosing may attenuate the improvement in glucose homeostasis and loss of adiposity after exercise training, which can be partially mitigated by intermittent rapamycin dosing schedules. Collectively, these data support the notion to test whether concomitant exercise and rapamycin could be used in older mice to delay, slow, or prevent the age‐related loss of health and function.

## 5 Methods

### 5.1 Animal Use and Care/Ethical Approval

All animal procedures were performed in conformance with institutional guidelines and were approved by the Institutional Animal Care and Use Committee of the William S. Middleton Memorial Veterans Hospital and University of Wisconsin‐Madison. Female C57BL/6J mice were procured from the Jackson Laboratory (000664) at 18 weeks of age. Mice were acclimated to the animal research facility for 2 weeks before entering studies. All mice were singly housed, provided Purina Rodent Chow (5001) ad libitum, and provided enrichment to minimize stress. The animal room was maintained with a 12:12 h light–dark cycle at 23°C.

### 5.2 PoWeR Training

Progressive weighted wheel running (PoWeR) was used as previously published (Dungan et al. 2019). Briefly, 20‐week‐old female C57BL6/J mice (*n* = 50) were acclimated to unweighted running wheels for 7 days. Mice that did not engage in 1 week of unweighted wheel running were allocated to the sedentary group (sedentary + vehicle control [*n* = 14, 3×/week]). The remaining 36 mice were randomized to the following groups to match for running volume and body composition: PoWeR + vehicle (*n* = 12, 3×/week), PoWeR + frequent rapamycin (3×/week; *n* = 12), PoWeR + intermittent rapamycin (1×/week, *n* = 12). After 1 week of unweighted wheel running, sedentary mice did not have access to a running wheel and were housed in cages of similar size with a hut and bedding for enrichment. For PoWeR, 1‐g magnets were fixed to one side of an 11 cm diameter wheel to asymmetrically load. Weights were progressively added starting at 2 g until 6 g at week 6 as previously performed (Dungan et al. 2019) for a total of 8 weeks of weighted wheel running. The weekly average of daily wheel running distance (km/day) was measured by ClockLab Software (Actimetrics, Wilmette, IL).

### 5.3 Rapamycin Treatment

Rapamycin (LC Laboratories) was dissolved in ethanol and stored at −80°C. On injection days, rapamycin was thawed and mixed with filter‐sterilized solution of 5% PEG 400 and 5% Tween‐80 in 0.9% saline. Vehicle‐treated mice received equal parts ethanol without rapamycin in 5% PEG 400 and 5% Tween‐80 in 0.9% saline. Injection volume ranged between 70 and 90 uL with the intended rapamycin concentration at 2 mg/kg body weight. All mice received equal handling and injections, which occurred 3 days per week (Monday/Wednesday/Friday) between the hours of 8 a.m.–10 a.m. Mice treated with rapamycin 1×/week received rapamycin on Monday and vehicle on Wednesday and Friday.

### 5.4 Body Composition

Body composition using an EchoMRI 3‐in‐1 body composition analyzer was completed on all mice before and after the seventh week of PoWeR. Mice were placed into a restraint tube (no anesthesia) for < 2 min of total time, generating duplicate measures of fat free mass (FFM) and fat mass. Duplicate measures were averaged for final data use.

### 5.5 Graded Exercise Test

Graded exercise tests were performed on a Columbus 3/6 treadmill (Columbus Instruments, Columbus OH) before and after 8 weeks of PoWeR. Mice were acclimated for two consecutive days prior to testing. Acclimation consisted of mice sitting on a stationary treadmill for 3 min with the shock grid activated to (3 Hz and 1.5 mA). Next, the treadmill was inclined to 10° and a speed of 6 m/min for 5 min then progressively increased to 12 m/min for 5 more minutes for a total of 10 min of treadmill movement. A graded exercise test to exhaustion was performed following a previously published method for measuring VO_2_ max in mice (Petrosino et al. 2016). Exhaustion was defined as spending five consecutive seconds in contact or repeated contact with the shock grid demonstrating an inability to reengage treadmill running. For post‐training testing, mice completed one acclimation day prior to graded exercise testing. All acclimations and graded exercise tests were performed in the early dark phase approximately 30–90 min after the light–dark transition. The investigator was blinded to treatment groups during testing.

### 5.6 Insulin and Glucose Tolerance Tests

Insulin tolerance tests (ITT) were performed during the third week of PoWeR, 24 h after the last exercise bout and 24 or 72 h after the previous rapamycin dose for the frequent (3×/week) and intermittent (1×/week) rapamycin groups, respectively. Following a 2‐h daytime fast beginning at 8:00 a.m., mice were i.p. injected with insulin (0.75 U/kg bodyweight). Blood glucose was recorded from a tail vein bleed before (0 min) and 15, 30, 45, 60, and 90 min post injection. Blood glucose was measured using a Bayer Contour Blood glucose meter and glucose strips. The investigator was blinded to treatment groups during testing.

The glucose tolerance test (GTT) was performed during the eighth week of PoWeR, 24 h after the previous exercise bout and 24 or 72 h after the previous rapamycin dose for the frequent (3×/week) and intermittent (1×/week) rapamycin groups, respectively. Following an overnight fast (~16 h), mice were i.p. injected with glucose (2 mg/kg bodyweight). Blood glucose was recorded from a tail vein bleed before (0 min) and 15, 30, 45, 60, 90, and 120 min post injection. The investigator was blinded to treatment groups during testing.

### 5.7 Grip Strength

Grip strength was evaluated before and after the eighth week of PoWeR by measuring peak force production of all limbs (fore and hind) using a Grip Strength Meter (Columbus Instruments, Columbus, OH). Mice were placed on the horizontal grid connected to a force transducer. Mice were held by the base of the tail and, when the mouse fully gripped the grid, the mouse was pulled horizontally at a consistent speed until the grip was released. This test was repeated three times with 15 min between tests. Grip strength was measured as the highest grams force across the three attempts and expressed as absolute. Testing was performed by a blinded investigator in the early dark phase.

### 5.8 Euthanasia and Tissue Collection

Running wheels were locked for 24 h prior to euthanasia. Rapamycin was dosed 24 h prior to euthanasia in the frequent rapamycin group (3×/week) and 7 days prior to euthanasia in the intermittent group (1×/week) to represent the steady‐state conditions the mice would be experiencing during the intervention prior to the next dose. Euthanasia was performed via cervical dislocation following a submandibular puncture for blood collection in the morning following a ~2–3 h fast. Muscles from both legs (tibialis anterior [TA], soleus, and flexor digitorum longus [FDL]) and heart were rapidly excised, weighed, and prepared for future biochemical analyses as outlined in each section. Muscle weights were expressed relative to body weight. Each tissue was dissected by the same investigator to limit variability.

### 5.9 Muscle Immunohistochemistry

Soleus and FDL from the left leg were weighed, measured for muscle length, and submerged in optimum cutting temperature compound (OCT, Tissue‐Tek; Sakura Finetek, The Netherlands) at resting length and frozen in liquid nitrogen chilled isopentane. Immunohistochemical staining and imaging of muscle cross sections were performed as previously described (Elliehausen et al. 2025).

### 5.10 Immunoblotting

The tibialis anterior, FDL, and soleus were powdered, prepared, and immunoblotted as previously detailed (Elliehausen et al. 2025). Antibodies used were P‐rpS6 (S235/236) (#4858), rpS6 (#2217), P‐AKT (S473) (#4060), and AKT (#4961) all obtained from Cell Signaling Technologies.

### 5.11 Statistical Analysis

Data in bar graphs presented as mean with individual data points. Data in line graphs are presented as mean with error bars expressed as standard error of the mean. One‐way ANOVA was used to compare the effects between Sedentary + Vehicle and PoWeR + Vehicle, PoWeR + Rapa 3×/week, and PoWeR + Rapa 1×/week. For comparisons with pre‐ and postmeasures, groups were compared with a two‐way ANOVA with repeated measures to determine the main effects of time, treatment, or an interaction. Both ANOVA approaches were *p* value adjusted by a two‐stage step‐up Benjamini Krieger Yukatelli false discovery correction rate with Q set to 5%. Discovery is denoted as an adjusted *p* value, *q* < 0.05. Before statistical analyses were performed, data were searched for outliers and removed via Grubbs outlier testing (alpha = 0.05). All statistical analyses were performed in GraphPad Prism (v10.4.1) with each statistical test described in figure legends.

## Author Contributions

Conceptualization and research design: C.J.E., A.R.K. Methodology: C.J.E., S.S.O., D.M.M., C.G.S., A.R.A., M.E.T., R.B. Formal analysis, writing – original draft: C.J.E., A.R.K. Writing – reviewed and edited: C.J.E., S.S.O., D.M.M., C.G.S., A.R.A., M.E.T., R.B., T.A.H., D.W.L., A.R.K. Resources: A.R.K., T.A.H. Funding acquisition: A.R.K.

## Conflicts of Interest

D.W.L. has received funding from, and is a scientific advisory board member of, Aeovian Pharmaceuticals, which seeks to develop novel, selective mTOR inhibitors for the treatment of various diseases.

## Supporting information


**Figure S1.** Transient inhibition of rpS6 following rapamycin administration. 5‐month‐old female C57BL6/J mice were treated with vehicle or rapamycin (i.p. 2 mg/kg bw^−1^, 1×/week) for 4 weeks. After a 4 h fast, insulin (i.p. 0.75 U/kg bw^−1^) stimulated muscle (tibialis anterior) was collected at 24, 48, or 72 h following the last rapamycin dose. Immunoblotting for phosphorylated (S235/236) and total rpS6 with a representative image. Data expressed as the ratio of phosphorylated to total protein. *N* = 9‐vehicle, 5–6 per timepoint post rapamycin injection. Data presented as mean plus individual data points. Data were analyzed for vehicle versus each timepoint by one‐way ANOVA with no corrections for multiple comparisons (Fisher’s LSD). *p < 0.05, **p < 0.01.
**Figure S2.** Cumulative running volume does not correlate with most dependent outcomes. Correlations of total wheel running volume versus various dependent outcomes. (A) Delta (post minus pre) adiposity, (B) glucose tolerance test area of the curve, (C) delta insulin tolerance test area under the curve, (D) normalized (muscle wet weight per bodyweight) soleus mass, (E) normalize FDL mass, (F) soleus type IIA myofiber average cross‐sectional area. Pearson *r* correlations tested on 34–36 mice per dependent outcome. *p* < 0.05 deemed significant.

## Data Availability

Data available upon reasonable request.
