# Supplementary material for: Rapamycin Does Not Compromise Exercise‐Induced Muscular Adaptations in Female Mice
Source: Aging Cell. 2025 Jul 24;24(10):e70183. doi: 10.1111/acel.70183 (PMC12507417; doi:10.1111/acel.70183)
Supplement: Supplementary file 1 — Figure S1. Transient inhibition of rpS6 following rapamycin administration. 5‐month‐old female C57BL6/J mice were treated with vehicle or rapamycin (i.p. 2 mg/kg bw−1, 1×/week) for 4 weeks. After a 4 h fast, insulin (i.p. 0.75 U/kg bw−1) stimulated muscle (tibialis anterior) was collected at 24, 48, or 72 h following the last rapamycin dose. Immunoblotting for phosphorylated (S235/236) and total rpS6 with a representative image. Data expressed as the ratio of phosphorylated to total protein. N = 9‐vehicle, 5–6 per timepoint post rapamycin injection. Data presented as mean plus individual data points. Data were analyzed for vehicle versus each timepoint by one‐way ANOVA with no corrections for multiple comparisons (Fisher’s LSD). *p < 0.05, **p < 0.01. Figure S2. Cumulative running volume does not correlate with most dependent outcomes. Correlations of total wheel running volume versus various dependent outcomes. (A) Delta (post minus pre) adiposity, (B) glucose tolerance test area of the curve, (C) delta insulin tolerance test area under the curve, (D) normalized (muscle wet weight per bodyweight) soleus mass, (E) normalize FDL mass, (F) soleus type IIA myofiber average cross‐sectional area. Pearson r correlations tested on 34–36 mice per dependent outcome. p < 0.05 deemed significant. [file ACEL-24-e70183-s001.docx]

**Supplementary Information**

**Rapamycin does not compromise exercise-induced muscular adaptations in female mice**

**Authors:** Christian J. Elliehausen^1,2^, Szczepan S. Olszewski^1,2^, Dennis M. Minton^1,2^, Carolyn G. Shult^1,2^, Aditya R. Ailiani^1,2^, Michaela E. Trautman^1,2^, Reji Babygirija^3,4^, Dudley W. Lamming^3,4,5^, Troy A. Hornberger^5,6,7^, Adam R. Konopka^1,2,5^

**Affiliations:** ^1^Division of Geriatrics and Gerontology, Department of Medicine, University of Wisconsin-Madison; ^2^Geriatric Research Education and Clinical Center, William S. Middleton Memorial Veterans Hospital; ^3^Division of Endocrinology, Department of Medicine, University of Wisconsin-Madison; ^4^William S. Middleton Memorial Veterans Hospital; ^5^University of Wisconsin-Madison Comprehensive Diabetes Center; ^6^Department of Comparative Biosciences, University of Wisconsin-Madison; ^7^School of Veterinary Medicine, University of Wisconsin-Madison


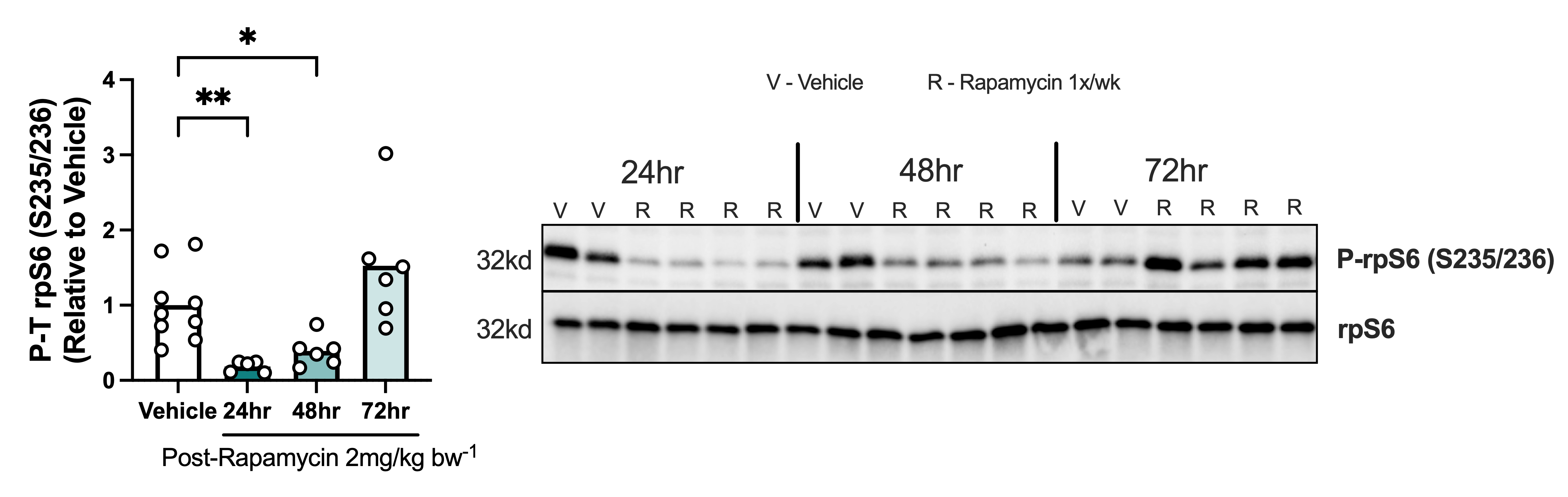


Figure S1. **Transient inhibition of rpS6 following rapamycin administration**. 5-month old female C57BL6/J mice were treated with vehicle or rapamycin (i.p. 2mg/kg bw^-1^, 1x/wk) for 4 weeks. After a 4 hour fast, insulin (i.p. 0.75U/kg bw^-1^) stimulated muscle (tibialis anterior) was collected at 24, 48, or 72-hr following the last rapamycin dose. Immunoblotting for phosphorylated (S235/236) and total rpS6 with a representative image. Data expressed as the ratio of phosphorylated to total protein. N=9-vehicle, 5-6 per timepoint post rapamycin injection. Data presented as mean plus individual data points. Data were analyzed for vehicle versus each timepoint by one-way ANOVA with no corrections for multiple comparisons (Fisher’s LSD). *P<0.05, **P<0.01.


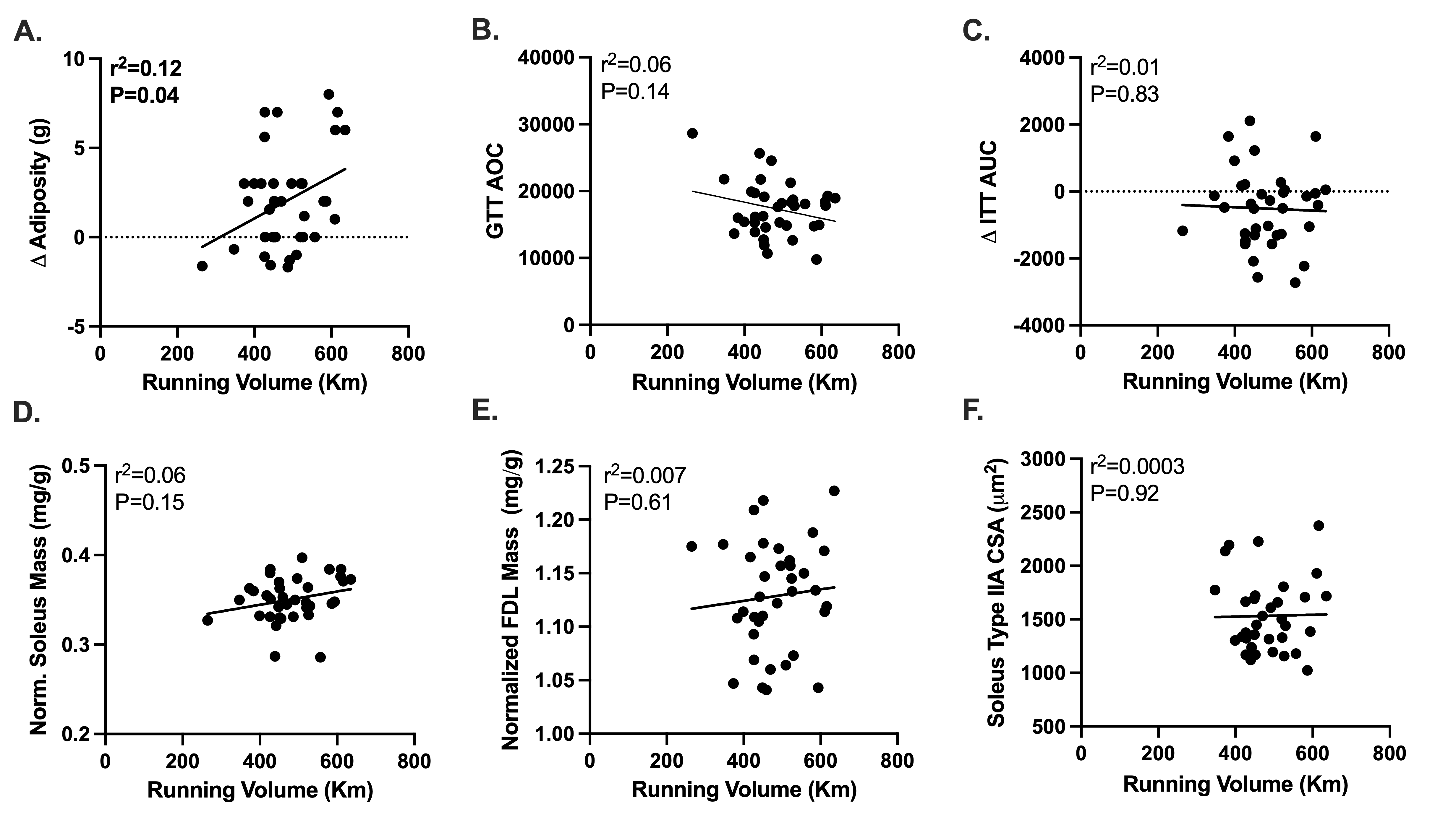
Figure S2: **Cumulative running volume does not correlate with most dependent outcomes.** Correlations of total wheel running volume versus various dependent outcomes. **(A)**  Delta (post minus pre) adiposity, **(B)** Glucose tolerance test area of the curve, **(C)** Delta insulin tolerance test area under the curve, **(D)** Normalized (muscle wet weight per bodyweight) soleus mass, **(E)** normalize FDL mass, **(F)** Soleus type IIA myofiber average cross-sectional area. Pearson r correlations tested on 34-36 mice per dependent outcome. P<0.05 deemed significant.
